# Genomics and Immunomics in the Treatment of Urothelial Carcinoma

**DOI:** 10.3390/curroncol29050283

**Published:** 2022-05-12

**Authors:** Veronica Mollica, Francesco Massari, Alessandro Rizzo, Roberto Ferrara, Arjun K. Menta, Jacob J. Adashek

**Affiliations:** 1 Medical Oncology, IRCCS Azienda Ospedaliero-Universitaria Di Bologna, 40138 Bologna, Italy; veronica.mollica2@unibo.it (V.M.); francesco.massari@aosp.bo.it (F.M.); 2 Struttura Semplice Dipartimentale di Oncologia Medica per la Presa in Carico Globale del Paziente Oncologico ‘Don Tonino Bello’, I.R.C.C.S. Istituto Tumori ‘Giovanni Paolo II’, Viale Orazio Flacco 65, 70124 Bari, Italy; a.rizzo@oncologico.bari.it; 3 Medical Oncology Department, Fondazione Istituto di Ricovero e Cura a Carattere Scientifico (IRCSS) Istituto Nazionale dei Tumori, 20133 Milan, Italy; robertoferrara86@gmail.com; 4 Molecular Immunology Unit, Department of Research, Fondazione Istituto di Ricovero e Cura a Carattere Scientifico (IRCSS) Istituto Nazionale dei Tumori, 20133 Milan, Italy; 5 Johns Hopkins University School of Medicine, Johns Hopkins University, Baltimore, MD 21205, USA; arjunkmenta@gmail.com; 6 Department of Internal Medicine, University of South Florida, Tampa, FL 33620, USA

**Keywords:** genomics, next-generation sequencing, urothelial cancer, immunotherapy

## Abstract

Urothelial carcinoma is a complex cancer with genomic immunomic drivers that have prognostic and predictive treatment implications. Identifying potential targetable alterations via next-generation sequencing and RNA sequencing may allow for elucidation of such targets and exploitation with targeted therapeutics. The role of immunotherapy in treating urothelial carcinoma has shown benefit, but it is unclear in which patients immunotherapeutics have the highest yield. Continuing efforts into better identifying which patients may benefit most from targeted therapies, immunotherapies, and combination therapies may ultimately lead to improved outcomes for patients with this disease.

## 1. Introduction

### Overview of Genomic Landscape of Urothelial Carcinoma

Urothelial carcinoma (UC) is a common tumor with approximately 160,000 new diagnoses and 31,000 estimated deaths annually [1]. Metastatic UC of the bladder or upper tract continues to represent a therapeutic oncologic challenge since an initial response to platinum-based chemotherapy, relapses, and disease progressions are common and difficult to manage. Immune checkpoint inhibitors (ICIs) radically changed patients’ outcomes, becoming the treatment of choice after progression to first-line chemotherapy or as maintenance therapy after stable disease (SD) or partial response (PR) to upfront platinum-based therapy [2]. Nonetheless, a consistent percentage of patients become resistant to these therapies and, in these cases, prognosis is grim with currently available treatment options. The genomic knowledge on UC is constantly growing and has opened up novel promising therapeutic approaches directed specifically towards tumor-specific molecular alterations. Whole genome and next-generation sequencing (NGS) has allowed a better characterization of the molecular pathways altered in UC that have led not only to carcinogenesis events, but also resistance to therapies and progression of disease [3]. The genomic profiling of UC aims at tailoring the oncological approach on the specific characteristics of the single patient, using druggable mutations to achieve a precision medicine treatment [4,5]. The Cancer Genome Atlas (TCGA) project for bladder cancer [6,7] reported a comprehensive overview of the genomic landscape of chemotherapy-naïve muscle-invasive bladder cancer (MIBC). Among the relevant findings of the 2014 TGCA analysis, MIBC resulted in a high somatic mutation rate (median 5.5/megabase) and genomic alterations in 69% of cases [6]. Of these, 44% of mutations were found on tyrosine kinase receptor/MAPK pathway, including fibroblast growth factor receptor (*FGFR*) in 17% of cases, human epidermal growth factor receptors (*EGFR*/*ERBB*) in 24% of cases, and 42% on phosphatidyl-inositol-3-kinase (*PI3K*)/*AKT*/mammalian target of rapamycin (*mTOR*) pathway. The subsequent 2017 TCGA analysis included 412 MIBC and pointed out 32 additional mutated genes, including *TP53*, *ARID1A*, *KMT2D*, and *KDM6A* [7]. Moreover, the two TGCA analyses described different cancer subtypes. The 2014 TGCA cohort identified luminal, luminal-infiltrated, basal, and squamous groups; conversely, the 2017 cohort divided MIBCs into five subgroups based on different RNA expression: basal squamous (35%), luminal papillary (35%), luminal-infiltrated (19%), luminal (6%), and neuronal (5%). Furthermore, a recent analysis by Kamoun et al. based on 1750 MIBC transcriptomic profiles identified six molecular classes with the aim of achieving a unanimous international consensus: basal/squamous (35%), luminal papillary (24%), luminal unstable (15%), stroma-rich (15%), luminal non-specified (8%), and neuroendocrine-like (3%) [8]. Dividing MIBC into different subclasses has the purpose of recollecting tumors with specific histologic, genomic, and microenvironment characteristics that could reflect clinical outcomes and guide a more precise oncologic therapeutic approach [9]. For example, a treatment repercussion could be correlated to the higher expression of *FGFR3* mutations and translocations of the luminal papillary subtype, thus making this group a good candidate for FGFR inhibitors.

Another developing field of research in UC consists of DNA damage response (DDR) gene alterations [10]. DDR genes are of pivotal importance to provide correction of genetic insults to DNA material and preserve genomic integrity [11,12]. Malfunctions of DNA repair mechanisms, including base or nucleotide excision repair, single- or double-strand breaks, or mismatch repair lead to unrepaired DNA damage, genomic instability, and carcinogenesis. DDR gene alterations have been found in 3–12% of MIBC and involve genes such as *BRCA1/2*, *ATM*, *PALB2*, *ERCC2*, and *FANCD2* [13]. Of note, tumors presenting DDR gene mutations have an increased response to poly (ADP-ribose) polymerase inhibitors (PARPi) and to platinum-based chemotherapy [12].

An important aspect to take into account is tumor heterogeneity, which could result in a diversified population of tumor cells presenting different genomic and morphological characteristics and functional alterations [14]. Tumor heterogeneity can be divided into interpatient, intratumoral, intertumoral, and temporal. These types of heterogeneity can explain differences in treatment responses within the same tumor histology, among regions of the primary tumor, and between the primary and metastatic lesions or different metastatic sites. Tumor heterogeneity should be addressed in particular cases with limited therapeutic options because, in some patients, a re-biopsy of metastatic sites could open the field to novel therapies targeted on specific alterations or that could be evaluated for enrollment in clinical trials.

Furthermore, epigenetic alterations should also be evaluated. Among these, telomerase reverse transcriptase (TERT) promoter mutations are gaining increasing attention, considering their high frequency in UC of the bladder [15]. In addition to the potential diagnostic value, TERT promoter alterations seem to also have prognostic repercussions. In fact, a retrospective analysis on patients with advanced UC treated with ICI showed that patients with TERT promoter mutation presented with improved survival [16]. Further investigation on this matter is needed.

In this review, we aim to elucidate the current knowledge regarding different molecular pathways that are altered in UC, which could have treatment repercussions, and to present novel diagnostic or therapeutic fields under investigation. The diversification of UC into different subgroups with specific molecular characteristics and increased knowledge regarding immunotherapy responses, and the advances in the diagnostic tools, including genomic and immunologic markers, could help to better tailor the therapeutic approach for individual patients.

## 2. Implicated Pathways

### 2.1. FGFR

The FGFR family includes four receptors, and fibroblast growth factors (FGFs) represent the native ligands for this family of kinases [17]. FGFRs represent transmembrane receptor tyrosine kinases made of one intracellular split tyrosine kinase domain and three extracellular immunoglobulin-like domains [18]. Interestingly, 18 ligands have been identified (FGF1, FGF2, FGF3, FGF4, FGF5, FGF6, FGF7, FGF8, FGF9, FGF10, FGF16, FGF17, FGF18, FGF19, FGF20, FGF21, FGF22, and FGF23), acting as paracrine or autocrine factors [19]. From a molecular point of view, upon dimerization, FGFR activates downstream signaling, causing the subsequent activation of RAS/MEK/ERK and PI3K/AKT signaling pathways [20]. FGFR pathways are involved in cell proliferation, differentiation, growth, and survival; in addition, FGFR interacts with other angiogenic pathways, including vascular endothelial growth factor (VEGF), and thus, play a role in angiogenesis [21].

FGFRs are aberrantly activated in around 5–10% of all solid tumors; with regards to UC, FGFR aberrations have been reported in approximately 15–20% of patients with metastatic disease, with *FGFR* mutations mainly located in the FGF binding region and the transmembrane helix, and more rarely in the kinase domain [22,23]. Interestingly, different levels of *FGFR* alterations have been reported according to different disease stages, with some studies suggesting changes in the rate of *FGFR* mutations from localized to metastatic urothelial carcinoma, with FGFR alterations appearing to be more frequent in non-muscle invasive bladder cancer [24,25]. In addition, some studies have reported that *FGFR* alterations could be more common in luminal papillary malignancies, which present lower T-cell infiltration [26].

### 2.2. FGFR Trials and Outcomes

Recent years have witnessed the development of a wide number of FGFR inhibitors for urothelial carcinoma patients, with this malignancy recently emerging as a disease entity with actionable molecular targets, broadening the therapeutic options and helping to improve clinical outcomes beyond the limited available standard treatments [27]. Initially, these agents were non-selective FGFR inhibitors (e.g., ponatinib, lucitanib, dovitinib, derazantinib, etc.) presenting off-target toxicities and very low activity. For example, a phase II trial assessing dovitinib in metastatic UC patients previously treated with platinum-containing systemic chemotherapy reported no responses in *FGFR3*-mutated subjects [28,29]. More recently, several selective FGFR inhibitors have emerged and have been developed, including, among others, the approved erdafitinib, pemigatinib, infigratinib, and rogaratinib, with these agents reporting class-effect toxicities and important efficacy in other solid tumors, including intrahepatic cholangiocarcinoma [30,31].

Distinct FGFR inhibitors showed different signs of activity in UC clinical trials, where the FGFR 1–4 inhibitor erdafitinib highlighted a higher overall response rate (ORR) of 40% compared with other agents [32]. In particular, the landmark erdafitinib, open-label, phase II trial explored the role of erdafitinib monotherapy in 99 UC patients with *FGFR* mutations or gene fusions [33]. All the patients presented a history of disease progression following at least one course of systemic chemotherapy or within one year after neoadjuvant or adjuvant chemotherapy; prior immune checkpoint inhibitor treatment was allowed. Of note, the authors reported median progression-free survival (PFS) and median OS of 5.5 months and 13.8 months, respectively, with these findings leading to Food and Drug Administration (FDA) approval for metastatic UC patients with selected *FGFR2*/*FGFR3* gene alterations. In addition, not only was the confirmed response rate 40%, but SD was reported in an additional 39% of enrolled subjects. These results have been corroborated by the recently published final analysis of this study, highlighting consistent activity and a manageable safety profile for erdafitinib, which currently remains the only FGFR inhibitor approved for metastatic *FGFR3*-mutated UC progressing on first-line treatment [34]. The most frequently reported treatment-related adverse events included hyperphosphatemia, stomatitis, diarrhea, vision problems, and dry mouth. Treatment discontinuation due to adverse events was observed in 13% of patients. As previously stated, slightly inferior activity was reported for other FGFR inhibitors, such as pemigatinib (ORR 25%) and infigratinib (ORR 25%, median PFS 3.75 months) in patients with *FGFR3* alterations [35,36,37] (Table 1).

In addition, the findings provided by erdafitinib have prompted clinicians to consider the expanding role of FGFR inhibitors in earlier stages of disease, as well as combining these drugs with other anticancer agents and figuring out the mechanisms of resistance to FGFR inhibitors. Among these current and future challenges, the ongoing phase III THOR study comparing erdafitinib versus chemotherapy or the programmed death-1 (PD-1) inhibitor pembrolizumab in metastatic UC patients with *FGFR* gene alterations will shed light on this topic (NCT03390504). In particular, this trial has the potential to define the best second-line treatment (erdafitinib versus pembrolizumab) in patients with FGFR alterations.

Moreover, erdafitinib is being tested in combination with the anti-PD-1, cetrelimab, in the ongoing phase II NORSE trial (NCT03473743) in previously untreated cisplatin-ineligible patients harboring FGFR mutations or fusions [44]. In the 19 evaluable patients at early results data cut-off, the combination resulted in clinically meaningful responses (ORR 68%) and a safety profile consistent with erdafitinib alone (most frequent adverse events: hyperphosphatemia, stomatitis, and diarrhea) (Table 2).

Mechanisms of resistance to FGFR inhibitors may be correlated to selection tumor cell clones that acquire survival strategies under the pressure of FGFR-targeting therapies and become independent from this pathway. These mechanisms include mutations or amplifications of other proteins implicated in this signaling pathway, such as MET, RAS, and EGFR [21]. Moreover, tumor heterogeneity could be another explanation for the development of resistance: FGFR-independent clones in the tumor bed could support the lack of response, conversely in regards to FGFR-dependent ones, as revealed from studies in other tumor types [14,45].

### 2.3. mTOR

The PI3K/AKT/mTOR pathway is a hallmark of carcinogenesis, considering its pivotal role in regulating cell growth. Its alteration can lead to uncontrolled cell growth resulting in tumorigenesis, angiogenesis, and metastatic spreading [46,47,48]. The mTOR is a serine/threonine-protein kinase that forms the catalytic subunit of mTOR Complex 1 (mTORC1) and 2 (mTORC2) [49]. mTOR is regulated by AKT, which mediates the phosphorylation and inactivation of the tuberous sclerosis complex 1 and 2 (TSC1/TSC2), with release of Rheb inhibition and mTOR activation.

The TCGA analysis showed that PI3K/AKT/mTOR pathway is altered in 42% of UC and the included alterations were: PIK3CA point mutations in 17%, deletion or mutation of TSC1 or TSC2 in 9%, and overexpression of AKT3 in 10% of cases [6].

### 2.4. PI3K/AKT/mTOR Trials and Outcomes

Considering the pivotal role of PI3K/AKT/mTOR pathway in multiple tumors, several drugs have been developed to target this signaling cascade with divergent results [47,50,51].

The mTOR inhibitor everolimus has been tested in a single-arm, phase II trial (NCT00805129) in 45 patients with advanced UC that progressed after one to four cytotoxic agents [52]. There were 2 PR and 12 minor regressions, but the study did not meet its primary endpoint of 2-month PFS. With regards to safety, the co-primary endpoint toxicities of grade 3/4 were observed in 29 patients (64%).

The combination of everolimus with pazopanib, an anti-angiogenic tyrosine kinase inhibitor, has been tested in a phase I study with 19 patients affected by metastatic UC, pretreated with one to three lines of chemotherapy [38]. The study was prematurely terminated for slow accrual, but it showed some clinical benefit: ORR was 21%, with one complete response (CR), three PR, eight SD, a median PFS of 3.6 months, and a median OS of 9.1 months [38]. The four patients that achieved clinical benefit (CR, PR, and SD) exhibited mutations in *TSC1/TSC2* or *mTOR*. In regards to the safety profile, 94.7% of patients presented adverse events, in particular all-grade hypophosphatemia, diarrhea, fatigue, thrombocytopenia, and hyperglycemia (Table 1).

Moreover, the identification of *PTEN* loss was associated with resistance to treatment with everolimus, despite preclinical studies showing that this alteration appeared to facilitate PI3K/AKT activation and consecutive stimulation of the mTOR pathway. These data suggest the use of a combination approach with PI3K and mTOR inhibitors [53].

Other PI3K/mTOR inhibitors currently under investigation in clinical trials in patients affected by advanced UC are: sapanisertib (TAK-228): a TORC1 and TORC2 inhibitor under evaluation in a phase II trial (NCT03047213), including patients with metastatic UC with TSC1 or TSC2 mutation; and buparlisib: a pan-class I PI3K inhibitor being studied in a phase II trial (NCT01551030) in pretreated patients with activating alterations within the PI3K/AKT/mTOR pathway. Furthermore, several ongoing studies are investigating PI3K/mTOR inhibitors in combination with other treatment approaches, including immunotherapy: nivolumab, an anti-PD-1, plus nabrapamycin, a macrolide antibiotic rapamycin bound to nanoparticle albumin (NCT03190174), nivolumab plus IPI-549, a PI3K-gamma inhibitor (NCT03980041 MARIO-275), paclitaxel plus sapanisertib, an oral inhibitor of TORC1 and TORC2 (NCT03745911), and durvalumab plus vistusertib, an mTOR-TORC1/2 inhibitor (NCT02546661, BISCAY, arm E).

The BISCAY trial deserves particular note [54]. It is a phase I adaptive, biomarker-directed platform study with the aim of investigating the anti-PD-ligand-1 (PD-L1) durvalumab in combination with different agents, depending on the molecular alteration found in previously treated patients with advanced UC: AZD4547 (FGFR1-3 tyrosine kinase inhibitor) in patients with FGFR1-3 mutations or fusions, olaparib (PARP inhibitor) in tumors with or without DNA homologous recombination repair (HRR) deficiency, and vistusertib in those with *TSC1/2* and *RICTOR* gene mutations. The strength of this study has shown that a biomarker-directed platform is a feasible design, even though combination approaches of immunotherapy and targeted therapy did not improve efficacy in terms of response rates (ranging between 9 and 36% not reaching efficacy criteria for further examination), PFS, and OS (similar between combination arm and durvalumab monotherapy) (Table 2).

### 2.5. HER2

Human EGFR are tyrosine-kinase receptors implicated in cell growth and proliferation—they include EGFR (ErbB1), HER-2 (ErbB2), HER-3 (ErbB3), and HER-4 (ErbB4) [55]. HER2 has a unique mechanism of action since it is an orphan receptor that can pass the signal without the need to bind to a ligand. The amplification of *ErbB2* gene leads to increased levels of HER2 protein in the cell membrane, its homodimerization, and resulting constitutive activation. This process activates downstream pathways such as RAS/RAF/MEK/ERK and AKT/mTOR, consequently promoting cell proliferation and survival [56]. In UC, the *ERBB* gene was found to be altered in 24% of cases [6].

**Figure 1:**
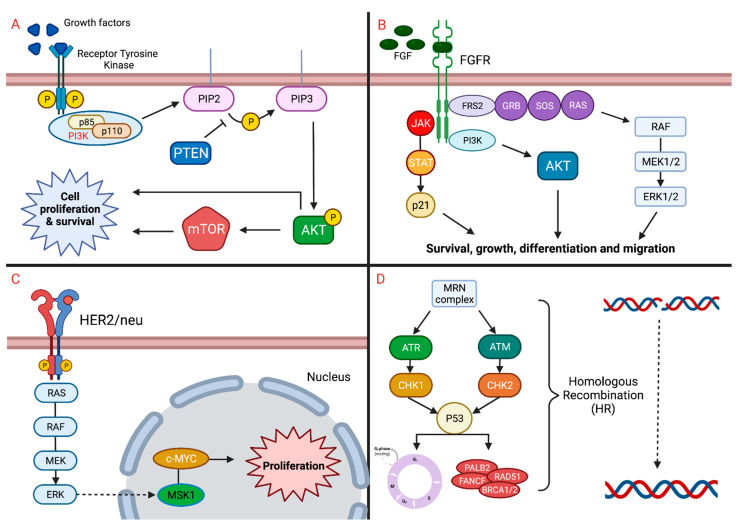
Various genomic pathways implicated in the pathogenesis of urothelial carcinoma. Panel (**A**) represents the PI3K, AKT, and mTOR pathway. Panel (**B**) represents the FGFR pathway and its involvement with the JAK/STAT, PI3K, and MAP kinase pathways. Panel (**C**) represents the HER2 pathway and its involvement with the MAP kinase pathway. Panel (**D**) represents the homologous recombination alterations and how they lead to pathogenesis of urothelial carcinoma.

HER2 has a relevant prognostic and predictive role in terms of response to targeted therapies in multiple types of cancer, including breast and gastric cancer [57]. Recently, the role of HER2 has also been investigated in UC, considering that mutations or amplifications of *ERBB2* gene have been identified in 9–12% of MIBC [6,7]. Furthermore, HER2 overexpression seems to have a prognostic role in patients with UC: in particular, it has been correlated with muscle-invasive disease, recurrence, shorter overall survival (OS), and more aggressive histologic variants, such as micropapillary or plasmacytoid subtypes [58,59,60]. It is still debated what the optimal method to assess HER2 expression is, considering the discordant results of fluorescence in situ hybridization (FISH), immunohistochemistry (IHC), and chromogenic in situ hybridization (CISH). In fact, FISH seems to reach lower positivity rates than IHC [59]. Figure 1 depicts the different pathways discussed (A. PI3K/AKT/mTOR; B. FGFR; C. HER2; D. DDR).

### 2.6. HER2 Trials and Outcomes

Several HER2-targeted agents have been investigated in the treatment of advanced UC patients with HER2 overexpression. Lapatinib is a reversible tyrosine-kinase inhibitor that targets and inhibits HER2 and EGFR, and blocks the activation of MAPK/extracellular signal-regulated kinases (ERK1/2), PI3K/AKT, and phospholipase C γ (PLCγ) downstream signaling pathways [61]. A phase III trial of maintenance therapy after first-line chemotherapy (four to eight cycles) investigated lapatinib compared with placebo in 232 patients with metastatic UC with HER1/2 overexpression [39]. The trial concluded with negative results, with the experimental arm not reaching a statistically significant difference compared to placebo in terms of median PFS (lapatinib 4.5 months versus placebo 5.1 months, hazard ratio [HR] 1.07, 95% confidence interval—CI 0.81–1.43, *p* = 0.63) and median OS (lapatinib 12.6 months and placebo 12.0 months, HR 0.96, 95% CI 0.70–1.31, *p* = 0.80) [39].

Trastuzumab is a humanized monoclonal antibody with the ability to inhibit intracellular signaling pathways through the binding of the extracellular domain IV of HER2 [62]. Moreover, it can activate antibody-dependent cell-mediated cytotoxicity by binding to the Fcγ receptor of natural killer cells. A single-arm phase II trial tested trastuzumab in combination with carboplatin, paclitaxel, and gemcitabine in chemotherapy-naïve patients with HER2 overexpression determined by IHC, gene amplification, or elevated serum Her-2/neu extracellular domain. The combination showed an ORR of 70% (31 of the 44 treated patients) with 5 CR and 26 PR, a median time to progression of 9.3 months, and median survival of 14.1 months. Nonetheless, the primary endpoint of cardiac toxicity was high, with grade 1 to 3 in 22.7% of cases, including one tachycardia and one left ventricular dysfunction (grade 3) [40]. Moreover, a randomized phase II trial investigated chemotherapy (platinum plus gemcitabine) alone or combined with trastuzumab in 61 chemotherapy-naïve patients with HER2 positivity determined with IHC (2+ or 3+) and FISH-positive results [63]. There was no statistically significant difference between the two arms in terms of median PFS (10.2 versus 8.2 months in the experimental and control arm, respectively, *p* = 0.689), ORR (65.5% versus 53.2%, *p* = 0.39), and median OS (15.7 versus 14.1 months, *p* = 0.684), presumably due to the low incidence of HER2 expression in the screened population. Nonetheless, an exploratory analysis suggested that patients in the trastuzumab arm that were treated with cisplatin presented with better outcomes than those treated with carboplatin (PFS 10.6 versus 8.0 and OS 33.1 versus 9.5 months, respectively).

In order to enhance the HER2 blockade, trastuzumab has also been evaluated in combination with pertuzumab, considering the positive results obtained in other types of tumor with HER2 overexpression, such as breast cancer. Considering that trastuzumab interferes with the ligand-independent HER2 signaling pathway, the dual HER2 inhibition has the rationale of also blocking the ligand-induced dimerization with the addition of pertuzumab, consisting in a humanized monoclonal antibody able to bind to domain II of HER2 that guides its dimerization with other Erb receptors. The phase IIa multiple basket MyPathway trial (NCT02091141) also included a cohort of 9 patients with platinum-resistant metastatic UC presenting HER2 amplification/overexpression [41]. This cohort of patients was treated with the combination of trastuzumab and pertuzumab, showing a certain grade of activity with an ORR of 33%, 1 CR, 2 PR, and 2 SD [41] (Table 1).

Trastuzumab deruxtecan is an antibody-drug conjugate (ADC) comprised of trastuzumab bound to a DNA topoisomerase 1 inhibitor payload with the ability to easily cross the cell membrane, thus also spreading the cytotoxic effect onto neighboring tumor cells not expressing the target. A phase I/II trial (NCT03523572) is testing trastuzumab deruxtecan in combination with nivolumab in patients pretreated with platinum-based chemotherapy with documented progression and with overexpression of HER2 determined with IHC (1+, 2+, or 3+). At the recent American Society of Clinical Oncology Genitourinary Cancer Symposium 2022 (ASCO-GU), the results of the primary analysis were presented as an abstract. At the data cut-off, 34 patients (30 in cohort 3, including patients with IHC 2+/3+, and 4 in cohort 4, enrolling patients with IHC 1+) received trastuzumab deruxtecan and nivolumab [64]. This combination presented promising antitumor activity in patients with high expression of HER2 (cohort 3: ORR 36.7%, median PFS was 6.9 months, and median OS 11.0 months). Of note, drug-related interstitial lung disease/pneumonitis, which is an adverse event of special interest for trastuzumab deruxtecan, occurred in 23.5% of patients, which is considered within the observed range with this compound in other monotherapy trials.

Afatinib is an irreversible oral inhibitor of the ERBB family. It has been tested in a phase II trial and demonstrated significant activity in patients with platinum-refractory UC with ERBB2 or ERBB3 alterations [65]. Other ongoing phase II trials are investigating afatinib in previously treated molecularly altered patients (NCT02795156) and in patients with ERBB1, ERBB2, and ERBB3 alteration (NCT02780687).

Moreover, among the novel compounds being evaluated there are: RC48-ADC, an ADC consisting of an anti-HER2 monoclonal antibody conjugated to a cytotoxic agent, and PRS-343, a bivalent, bispecific fusion protein made of an anti-HER2 monoclonal antibody bound to a CD137-targeting anticalin. RC48-ADC is under investigation in a phase II trial in HER2-negative (IHC 0 or 1+, NCT04073602) or HER2 overexpressed (IHC 2+ or 3+, NCT03809013) previously treated patients. PRS-343 is being tested in monotherapy in a phase I trial (NCT03330561) and in combination with atezolizumab in another phase I study (NCT03650348) in pretreated HER2-positive solid tumors including UC patients (Table 2).

### 2.7. DDR Trials and Outcomes

The preclinical evidence showing the presence of DDR gene alterations in UC and the promising results of PARP inhibitors in tumors presenting these mutations also led to the investigation of these compounds in patients with UC. A report on two patients with UC presenting DDR gene mutations (germline mutation in *BRCA1* and *CHEK2* in one patient and somatic loss-of-function mutation in *BRCA2* in the other) showed a good response to therapy with olaparib [66]. In particular, the increased number of somatic aberrations generated by DDR gene alterations can lead to an enhanced tumor mutational burden and neoantigen release, thus eliciting a more immunogenic tumor profile. These factors suggest a higher probability of response to immunotherapy.

### 2.8. DDR Trials and Outcomes

Olaparib is under investigation in a phase II study in patients with DDR gene alterations and cisplatin-ineligible untreated or progressed to first-line chemotherapy (NCT03448718). Another phase II trial is testing olaparib in pretreated patients presenting DDR gene mutations (NCT03375307). Furthermore, a phase II study is evaluating the combination of olaparib and AZD6738 (an inhibitor of ataxia telangiectasia and rad3-related kinase) in later lines of therapy (NCT03682289).

The PARP inhibitor rucaparib has been tested in the ATLAS trial, a phase II study that enrolled 97 pretreated metastatic UC patients independently of homologous recombination deficiency (HRD) status [67]. The primary endpoint was ORR in the intent-to-treat (ITT) and HRD-positive (corresponding to 20.6% of enrolled patients) population. The results of the trial showed that rucaparib did not present significant activity independently from HRD status, with no confirmed responses in the ITT population.

The PARP inhibitor niraparib is being evaluated in an ongoing phase I–II study (NCT03425201) in combination with the tyrosine kinase inhibitor cabozantinib in patients with metastatic UC progressed to platinum-based chemotherapy. The rationale of this combination lies in the inhibitory activity of cabozantinib on c-Met, that, when overexpressed, seems to be able to decrease response to PARP inhibitors. Moreover, a randomized phase II trial is testing niraparib alone as maintenance therapy in patients achieving a response to first-line platinum-based chemotherapy not selected for DDR status (NCT03945084).

Considering the interplay between DDR genes and several factors able to stimulate the immune response, the combination of PARP inhibitors and PD-1/PD-L1 blockade is a promising treatment approach [68]. A study by Teo et al. recollected patients with metastatic UC enrolled in prospective clinical trials treated with atezolizumab or nivolumab (NCT02553642, NCT01928394, and NCT02108652) [69] and aimed at examining the relationship between DDR gene alterations and treatment response. Of the 60 patients that met the eligibility criteria, DDR gene mutations were identified in 43 patients (72%). The presence of these mutations resulted in being independently associated with immunotherapy response, and in particular, the response rate was 67.9% in mutated patients versus 18.8% in those without any alteration (*p* < 0.001). Nonetheless, as already pointed out, the arm of the BISCAY trial investigating durvalumab plus olaparib failed to show the benefit of the combination, with a response rate in the 14 patients selected for HRR deficiency of 35%, and in the 22 not selected for those alterations of 9%. In addition, the combination of niraparib plus atezolizumab is currently under evaluation in a phase I–II trial in patients with metastatic UC progressed to one platinum-containing regimen (MORPHEUS-UC, NCT03869190).

Furthermore, a phase Ib–II trial (SEASTAR, NCT03992131) is investigating the combination of rucaparib with other agents in metastatic pretreated UC patients. In particular, rucaparib has been associated with lucitanib (a VEGFR1-2-3, FGFR1-2, and PDGFRα-β inhibitor) and sacituzumab govitecan (a humanized anti-trophoblast cell surface antigen-2, Trop2, monoclonal antibody IgG1 bound to SN-38) in a phase Ib–II trial (SEASTAR, NCT03992131).

The ATLANTIS adaptive, multicomparison, phase II trial compared maintenance rucaparib to placebo in HRD-positive patients that did not progress to 4–8 cycles of platinum-based chemotherapy. Patients were selected based not only on the presence of somatic or germline alterations in HRD genes (panel of 15 genes), but also based on ≥10% genomic loss of heterozygosity (% LOH). Maintenance rucaparib extended PFS in biomarker-selected patients, with a median PFS of 35.3 weeks compared to 15.1 weeks of placebo (HR 0.53, 80% CI 0.30–0.92, 1-sided *p* = 0.07) [70].

The randomized phase II BAYOU trial evaluated the combination of durvalumab plus olaparib or matching placebo in first-line treatment of platinum-ineligible patients (HRR mutant and wild-type). The study did not meet the primary endpoint of PFS in the intention-to-treat population, but there seems to be a positive trend in HRR-mutated patients (median PFS 5.6 months in the experimental group and 1.8 months in the control group) [71] (Table 2).

### 2.9. Antibody-Drug Conjugate

ADCs are an innovative field that is gaining increasing attention and deserves a separate mention. This treatment approach finds its rationale in combining a cytotoxic agent with a targeted therapy directed against tumor-associated antigens with the aim to restrict the field of action to tumor cells expressing those particular markers on their surface, thus reducing systemic exposure and toxicity.

In UC, two ADCs are gaining increasing attention. Sacituzumab govitecan is composed of SN-38, an active metabolite of the cytotoxic agent irinotecan (a topoisomerase 1 inhibitor), and an anti-Trop2, a transmembrane glycoprotein highly expressed on epithelial cancer cells surface, including UC cells. The cohort 1 of the multicohort phase II TROPHY-U-01 trial (NCT03547973) enrolled 113 patients with advanced or metastatic UC progressed to platinum-based chemotherapy and ICI to receive sacituzumab govitecan [42]. The primary endpoint of ORR resulted in being 27%, with median duration of response of 7.2 months, median PFS of 5.4 months, and median OS of 10.9 months, thus making this treatment an extremely promising option for pretreated patients. Of note, among grade 3–4 treatment-related adverse events, there are: neutropenia (35%), leukopenia (18%), anemia (14%), diarrhea (10%), and febrile neutropenia (10%). Furthermore, at ASCO-GU 2022, the interim results of cohort 3 of TROPHY-U-01 trial were presented (on 41 patients that received at least a dose of sacituzumab govitecan at the time of data cut-off) [72]. In this cohort, patients received sacituzumab govitecan combined with pembrolizumab as second-line treatment after progression to platinum-based chemotherapy. This combination showed promising ORR (34%) and clinical benefit rate (44%) with a manageable safety profile, supporting further evaluation (Table 1).

Enfortumab vedotin is another ADC composed of an anti-nectin-4 fully human monoclonal antibody conjugated to monomethyl auristatin E (MMAE), a microtubule-disrupting agent. Nectin-4 is a transmembrane adhesion protein expressed in up to 60% of UC, with limited expression in normal tissues, and only 25–30% of its sequence is identical to other receptors of the nectin family, thus making it a good candidate to restrict its field of action [73]. The phase III randomized EV-301 trial tested enfortumab vedotin compared to investigator-chosen chemotherapy (docetaxel, paclitaxel, or vinflunine) in 608 patients with metastatic UC previously treated with platinum-based chemotherapy and who had disease progression during or after treatment with a PD-1/L1 inhibitor [43]. The primary endpoint, ORR, was 52%, with 20% of CR and 31% of PR [43]. In addition, PFS was also increased with a median PFS of 5.55 months in the enfortumab vedotin group and 3.71 months in the chemotherapy group (HR = 0.62, 95% CI 0.51–0.75; *p* < 0.00001). These results make enfortumab vedotin a valid treatment option in pretreated patients. Treatment-related adverse events were similar between the two groups, with all grade and ≥3 grade adverse events rates of 93.9% and 51.4% in the experimental arm, respectively, and 91.8% and 49.8% in the chemotherapy arm, respectively. Among the frequent adverse events of enfortumab vedotin there are: alopecia (45.3%), peripheral sensory neuropathy (33.8%), pruritus (32.1%), fatigue (31.1%), decreased appetite (30.7%), diarrhea (24.3%), dysgeusia (24.3%), and nausea (22.6%). In addition, the phase 2, single-arm EV-201 study investigated enfortumab vedotin in 91 cisplatin-ineligible patients with metastatic UC previously treated with PD-1/L1 inhibitors [74]. Grade 3 or worse treatment-related adverse events were reported in 55% of patients and included: neutropenia (9%), maculopapular rash (8%), and fatigue (7%). Four treatment-related deaths were reported (acute kidney injury, metabolic acidosis, multiple organ dysfunction syndrome, and pneumonitis). Considering the limited treatment options for cisplatin-ineligible patients, the results of this trial suggest that enfortumab vedotin could be a promising alternative.

The ongoing multicohort EV-103 trial (NCT03288545) is testing the combination of enfortumab vedotin in first-line treatment of cisplatin-ineligible patients. The updated results of cohort A (dose escalation) on 45 patients confirmed the promising activity of this combination, with an ORR of 73.3% (17.8% complete response) and a manageable safety profile, with peripheral sensory neuropathy (56%), fatigue (51%), and alopecia (49%) as the most common side effects [75].

It needs to be noted that the best treatment sequence for patients progressing to first-line chemotherapy and/or immunotherapy is still unknown. In fact, FGFR inhibitors and ADC are promising therapeutic strategies that are opening up new fields to pretreated patients, but there is still a lot to understand in regards to treatment sequencing and development of resistance. Nonetheless, as already stated, the current FDA approval for erdafitinib is for patients with *FGFR2/FGFR3* alterations progressed to platinum-based chemotherapy, while sacituzumab govitecan and enfortumab vedotin have been granted approval for patients also pretreated with a subsequent line of treatment with an anti-PD-1/PD-L1.

## 3. Immunotherapy in Urothelial Carcinoma

### 3.1. Immune Checkpoint Inhibitors

The benefit of adding immunotherapy to the treatment landscape is evidenced in multiple settings, primarily in platinum-ineligible patients and in the adjuvant setting. In the frontline setting, atezolizumab, an anti-PD-L1 antibody, has been shown to yield significant responses and survival advantages for patients [76,77]. This was particularly the case in patients whose tumor expressed PD-L1 or, regardless of PD-L1 status, were platinum-ineligible [76,77]. In this patient population, the ORR was 23%, with a 9% CR rate and a median duration of response that was not reached at 17.2 months of follow-up [77]. The median PFS in this study was 2.7 months and median OS was 15.9 months [77]. For patients whose tumors had PD-L1 expression or those ineligible to receive platinum-based chemotherapy, this provides a valuable treatment option.

Interestingly, the PD-1 inhibitor, pembrolizumab, showed no improvement in median PFS, 8.3 versus 7.1 months (HR 0.78, 0.65–0.93; *p* = 0.0033) or median OS, 17 versus 14.3 months (HR 0.86, 0.72–1.02; *p* = 0.0407) when given in combination with standard chemotherapy in the frontline setting [78]. However, when compared to those who only received pembrolizumab versus standard chemotherapy, the survival rates were similar, 15.6 months for those receiving pembrolizumab versus 14.3 months in patients who received chemotherapy, which has been interpreted to mean that pembrolizumab could offer potential treatment options for those patients who are platinum-ineligible and thus it could become incorporated in the National Comprehensive Cancer Network guidelines [79].

Of note, in the first-line setting, atezolizumab and pembrolizumab are approved for PD-L1-positive patients, but with different assessing methods and cut-offs. For pembrolizumab, in KEYNOTE-052, PD-L1 was determined using the IHC 22C3 pharmDx assay (Agilent Technologies, Carpinteria, CA, USA) with a cut-off for combined positive score (CPS) of ≥10%. Conversely, with regards to atezolizumab (IMvigor210 trial), PD-L1 expression was evaluated on tumor-infiltrating immune cells (IC) with VENTANA SP142 IHC assay (Ventana Medical Systems, Inc.; Tucson, AZ, USA) and the scoring was IC0, IC1, or IC2/3 for PD-L1 expression on <1%, ≥1% and <5% or ≥5% of IC, respectively.

With regards to second-line treatment in patients progressed to platinum-based chemotherapy, pembrolizumab has been approved on the basis of the results of the randomized phase III KEYNOTE-045 trial [80]. Pembrolizumab compared to investigators’ choice chemotherapy resulted in being associated with an improved median OS (10.3 months versus 7.4 months, respectively, HR 0.73, 95% CI 0.59–0.91; *p* = 0.002) and higher ORR (21.1% versus 11.4%, respectively). The phase II IMvigor210 [76] and the phase III IMvigor211 [81] trials investigated atezolizumab in patients pretreated with platinum-based chemotherapy. While the IMvigor210 cohort 2 presented promising results with an ORR of 15%, the subsequent randomized IMvigor211 trial failed to show a benefit in OS (primary endpoint in patients with PD-L1 expression ≥ 5%) of the experimental arm compared to chemotherapy of the investigators’ choice (median OS 11.1 months in atezolizumab group versus 10.6 months in chemotherapy group, respectively, HR 0.87, 95% CI 0.63–1.21; *p* = 0.41). Moreover, nivolumab has been tested in second-line treatment after platinum-based chemotherapy in the phase II CheckMate 275 trial [82]. The anti-PD-1 was demonstrated to have good antitumoral activity, with an ORR of 20% in the overall population and 28.4% in patients with PD-L1 expression ≥5%. To improve these results, nivolumab has been tested in combination with ipilimumab in the TITAN-TCC trial (NCT0321977) [83].

Recently, at ASCO-GU 2022, the results of cohort 2 have been presented (nivolumab 1 mg/kg plus ipilimumab 3 mg/kg boost, 2–4 doses in non-responder patients to 4 doses of nivolumab 240 mg every 2 weeks after platinum-based chemotherapy). The primary endpoint of ORR resulted in being 32.5% in the overall population and 46% in patients with PD-L1 ≥1%. Double immunotherapy has also been evaluated in the phase III DANUBE trial, which randomized 1032 previously untreated patients with advanced UC to receive durvalumab monotherapy, durvalumab plus tremelimumab, or platinum-based chemotherapy [84]. The study did not meet the co-primary endpoints of OS in the durvalumab monotherapy versus chemotherapy groups in high PD-L1 patients (14.4 months versus 12.1 months, respectively, HR 0.89, 95% CI 0.71–1.11; *p* = 0.30) and between the durvalumab plus tremelimumab versus chemotherapy groups in the overall population (15.1 months versus 12.1 months, respectively, HR 0.85, 95% CI 0.72–1.02; *p* = 0.075).

In the adjuvant or maintenance setting, immune checkpoint inhibitors have also yielded clinical benefit for patients [85]. The JAVELIN Bladder 100 trial randomized 700 patients to receive either maintenance avelumab, a PD-L1 inhibitor, or placebo in patients achieving a stable disease or partial response to first-line platinum-based chemotherapy. In patients who received avelumab, the median OS was 21.4 versus 14.3 months in those who received placebo (HR 0.69; 0.56–0.86; *p* = 0.001) [86].

With regards to the neoadjuvant setting, several phase II trials investigated ICIs in cisplatin-ineligible patients [87]: atezolizumab in ABACUS trial (pathological complete response—pCR 31%) [88], pembrolizumab in PURE-01 trial (pCR 41%) [89], durvalumab plus the CTLA-4 inhibitor tremelimumab (pCR 37.5%) [90], and nivolumab plus ipilimumab in the NABUCCO study (pCR 46%) [91].

A similar study in the same adjuvant setting, CheckMate 274, compared nivolumab to placebo in 709 patients [92]. In patients who received adjuvant nivolumab, the median disease-free survival was 20.8 versus 10.8 months for those who received placebo. The median recurrence-free survival was 22.9 versus 13.7 months for those patients who received nivolumab versus placebo, respectively [92]. This served as an additional trial, yielding clinically meaningful benefits for patients in the maintenance setting [93].

Many of these studies investigated the role of PD-L1 expression and response rates. This becomes more important in the treatment decision process for patients who are cisplatin-eligible but may have other comorbidities precluding them from platinum chemotherapies or where immune checkpoint inhibitors may be more ideal. Roughly 24–38% of invasive UC is found to be genomically unstable and may account for the positive responses seen in patients with UC who receive immune checkpoint inhibitors [94,95].

Different clinical trials have shown that besides PD-L1, multiple biomarkers characterizing tumor microenvironment such as TMB and T-cell infiltration at GEP are potentially clinically useful to better select UC patients for treatment with PD-1/PD-L1 inhibitors [96].

### 3.2. Hyperprogressive Disease

An important and understudied area in patients with UC receiving immune checkpoint inhibitors is the phenomenon of developing hyperprogressive disease (HPD) [97,98]. In a study with 101 patients with UC, 6.4% (*n* = 13) were found to have HPD, which was defined as a greater than 2-fold increase in tumor growth rate, a greater than 50% increase in tumor burden, or development of extensive (10 or more) new lesions. This study also found that a 30% increase in lymphocyte number after receiving a PD-1/PD-L1 inhibitor was a negative predictor of HPD, and the median OS in patients who developed HPD was 3.5 months [99]. An additional study reported the response outcomes of 23 patients with UC who were given pembrolizumab [100]. Previous groups have described the impact of *EGFR* and *MDM2* alterations associating with HPD [101]. In the study of 23 patients, 26% (*n* = 6) were characterized as having HPD defined as a >50% increase in tumor burden compared with pre-immunotherapy imaging, a time-to-treatment failure < 2 months, and a >2-fold increase in progression pace [100]. In the 6 patients with HPD, squamous differentiation, which was also positive for EGFR, along with MAC387 expression, were the exclusive defining features and were not present in patients who responded to therapy (*p* = 0.0019) [100]. The role of HPD in UC is not well-understood and not well-characterized, but is clearly a legitimate phenomenon and concern in this patient population. More resources for identifying risks for development of HPD are important to better monitor these patients and improve their clinical management [102].

## 4. Future Directions and Conclusions

The advent of genomics and immunomics has impacted the treatment landscape substantially in patients with UC. Efforts continue to improve treatment strategies and surveillance strategies. Addition of circulating tumor DNA (ctDNA) testing NGS has allowed for potential early diagnostics as well as surveillance testing [103]. Ongoing efforts have shown that ctDNA testing has allowed for improved early initiation of therapies and improved disease-free survival when treated (HR 0.58, 0.43–0.79; *p* = 0.0024) [104]. This has also supported the use of surveillance where clearance of ctDNA in patients who were given adjuvant atezolizumab has improved survival (*p* = 0.0204) compared to those who continued to have ctDNA positivity [104]. Similarly, serial ctDNA testing was able to predict 90% of disease progression in patients at 6 months as well as durability of previous responses in those with aggregate variant allele frequencies of less than or equal to 0.7 in three consecutive samples [105]. Adding ctDNA to the therapeutic armamentarium for early detection as well as surveillance testing will likely continue to play a significant role in improved survival in patients with UC.

Continued efforts to integrate NGS into practice may also aid in guiding treatment decisions [106,107]. Using combination strategies to combine a patient’s specific tumor alterations and potentially immunomic expression may also yield benefit in improving patients’ outcomes with UC [5,108,109,110,111]. Customized n-of-1 approaches for treatments for patients may offer unique options for those otherwise unable to obtain population-based standard-of-care therapies such as platinum chemotherapies, for those who experience lack of benefit from immunotherapy, or in patients at high risk for developing HPD [112]. Further efforts into combining patient-level genomic and transcriptomic data may help identify meaningful alterations and allow drugs to effectively hit their targets [113,114,115]. The treatment landscape for patients with UC continues to move at an expeditious pace. Integrating diagnostic strategies such as NGS, ctDNA, and transcriptomics along with using valid combinations may ultimately lead to a continuation of improvement of outcomes for patients with UC.

## Figures and Tables

**Table 1:** Targeted therapies and outcomes.

Gene Alteration	Drug	Mechanism of Action	Number of Patients	Outcome	Reference
FGFR	Erdafitinib	Tyrosine kinase inhibitor of FGFR1–4	99	ORR 40% PFS 5.5 months OS 13.8 months	[34]
FGFR	Pemigatinib	Tyrosine kinase inhibitor of FGFR1-3	140 (Interim analysis: 100)	ORR 25%	[37]
FGFR	Infigratinib	Tyrosine kinase inhibitor of FGFR1-3	67	ORR 25% PFS 3.75 months OS 7.75 months	[36]
mTOR	Everolimus + pazopanib	Inhibitor of mTOR + inhibitor of VEGF	19	ORR 21% PFS 3.6 months OS 9.1 months	[38]
HER2	Lapatinib	Tyrosine-kinase inhibitor against HER2 and EGFR	232	PFS 4.5 months OS 12.6 months	[39]
HER2	Trastuzumab + carboplatin, paclitaxel, gemcitabine	Monoclonal antibody against HER2	44	PFS 9.3 months OS 14.1 months	[40]
HER2	Trastuzumab + pertuzumab	Monoclonal antibody against HER2	9	ORR 33%	[41]
Trop2	Sacituzumab govitecan	ADC of active metabolite of the cytotoxic agent irinotecan and transmembrane glycoprotein highly expressed on epithelial cancer cells surface	113	ORR 27% PFS 5.4 months OS 10.9 months	[42]
Nectin-4	Enfortumab vedotin	ADC of anti-nectin-4 conjugated to monomethyl auristatin E	608	ORR 52% PFS 5.55 months	[43]

ADC, antibody-drug conjugate; ORR, overall response rate, OS, overall survival, PFS, progression-free survival.

**Table 2:** Ongoing trials of targeted therapies.

Gene Alteration	Drug	Number of Patients Planned to Accrue	Primary Outcome	NCT Number
FGFR aberrations	Erdafitinib	631	OS	NCT03390504 (THOR)
TSC1/TSC2 mutations	Sapanisertib	209	ORR	NCT03047213
Unselected	Buparlisib	19	2-months PFS; PFS in the expansion cohort	NCT01551030
Unselected	Nivolumab + nabrapamycin	34	Maximum tolerated dose	NCT03190174
Unselected	Nivolumab + IPI-549	160	ORR	NCT03980041 (MARIO-275)
Unselected	Paclitaxel + sapanisertib	52	ORR	NCT03745911
HER2 overexpressed	Trastuzumab deruxtecan + nivolumab	99	Part 1: dose-limiting toxicity Part 2: ORR	NCT03523572
EGFR, HER2, VEGFR, FGFR1/2, MET	Afatinib Regorafenib Cabozantinib	100	ORR	NCT02795156
ERBB1, ERBB2, ERBB3	Afatinib	42	6-months PFS	NCT02780687
HER2-negative	RC48-ADC	19	ORR	NCT04073602
HER2-positive	RC48-ADC	60	ORR	NCT03809013
HER2-positive	PRS-343	85	Incidence and severity of adverse events	NCT03330561
HER2-positive	PRS-343 + atezolizumab	45	Incidence of dose-limiting toxicities; recommended phase 2 dose	NCT03650348
DDR genes	Olaparib	30	ORR	NCT03448718
DDR genes	Olaparib	60	ORR	NCT03375307
ARID1A, ATM	Olaparib + AZD6738	68	ORR	NCT03682289
Unselected	Niraparib + cabozantinib	20	Maximum tolerated dose; PFS	NCT03425201
Unselected	Niraparib	58	PFS	NCT03945084
Unselected	Durvalumab + olaparib	154	PFS	NCT03459846
Unselected	Atezolizumab + enfortumab vedotin; Atezolizumab + niraparib; Atezolizumab + Hu5F9-G4; Atezolizumab + tiragolumab; Atezolizumab + sacituzumab govitecan; Atezolizumab + tocilizumab; Atezolizumab + RO7122290	645	ORR	NCT03869190 (MORPHEUS-UC)
BRCA1, BRCA2, PALB2, RAD51C, RAD51D	Rucaparib + lucitanib; Rucaparib + sacituzumab govitecan	329	Phase 1b: Safety and tolerability; Dose-limiting toxicityPhase 2: ORR	NCT03992131 (SEASTAR)

ADC, antibody-drug conjugate; ORR, overall response rate, OS, overall survival, PFS, progression-free survival.
